# Per- and Polyfluoroalkyl Substances (PFAS) Mixture during Pregnancy and Postpartum Weight Retention in the New Hampshire Birth Cohort Study (NHBCS)

**DOI:** 10.3390/toxics11050450

**Published:** 2023-05-10

**Authors:** Yuting Wang, Caitlin Howe, Lisa G. Gallagher, Julianne Cook Botelho, Antonia M. Calafat, Margaret R. Karagas, Megan E. Romano

**Affiliations:** 1 Department of Epidemiology, The Geisel School of Medicine at Dartmouth, Lebanon, NH 03755, USA; 2 Division of Laboratory Sciences, Centers for Disease Control and Prevention, Atlanta, GA 30341, USA

**Keywords:** per- and polyfluoroalkyl substances, postpartum weight retention, mixture, prenatal exposure, endocrine disruptors, maternal and child health

## Abstract

Per- and polyfluoroalkyl substances (PFAS), widely used in industrial and consumer products, are suspected metabolic disruptors. We examined the association between a PFAS mixture during pregnancy and postpartum weight retention in 482 participants from the New Hampshire Birth Cohort Study. PFAS concentrations, including perfluorohexane sulfonate, perfluorooctane sulfonate (PFOS), perfluorooctanoate (PFOA), perfluorononanoate (PFNA), and perfluorodecanoate, were quantified in maternal plasma collected at ~28 gestational weeks. Postpartum weight change was calculated as the difference between self-reported weight from a postpartum survey administered in 2020 and pre-pregnancy weight abstracted from medical records. Associations between PFAS and postpartum weight change were examined using Bayesian kernel machine regression and multivariable linear regression, adjusting for demographic, reproductive, dietary, and physical activity factors; gestational week of blood sample collection; and enrollment year. PFOS, PFOA, and PFNA were positively associated with postpartum weight retention, and associations were stronger among participants with a higher pre-pregnancy body mass index. A doubling of PFOS, PFOA, and PFNA concentrations was associated with a 1.76 kg (95%CI: 0.31, 3.22), 1.39 kg (−0.27, 3.04), and 1.04 kg (−0.19, 2.28) greater postpartum weight retention, respectively, among participants who had obesity/overweight prior to pregnancy. Prenatal PFAS exposure may be associated with increased postpartum weight retention.

## 1. Introduction

The prevalence of obesity continues to rise in the United States, with approximately 40% of women of childbearing age classified as obese in 2020 [1]. Maternal obesity has been associated with a variety of adverse health outcomes for both mothers and offspring [2]. Individuals with a body mass index (BMI) ≥ 25 kg/m^2^ during pregnancy are more susceptible to gestational complications including pre-eclampsia and gestational diabetes, which further increase the risk of developing type II diabetes and cardiovascular diseases (CVDs) in later life [2,3]. In addition to associations with miscarriage, macrosomia, and preterm birth, maternal obesity has long-term effects on offspring health, such as increased risk of childhood obesity, obesity in adulthood, type II diabetes, and CVD [4]. Excessive gestational weight gain (GWG) and postpartum weight retention have been identified as important contributors to the development of obesity among women of reproductive age [5,6]. Additionally, although GWG is a key predictor of postpartum weight retention, independent associations between postpartum weight retention and future cardiovascular risk have been identified, even after adjusting for both maternal pre-pregnancy BMI and GWG [7]. To break the intergenerational cycle of obesity and improve maternal and child health, it is of public health importance to identify modifiable risk factors for obesity and develop targeted interventions to reduce weight retention in the postpartum period [6,8,9].

Per- and polyfluoroalkyl substances (PFAS) are a group of manufactured chemicals that are widely used to make products resistant to stains, oil, and water [10]. PFAS are widely applied in industrial and consumer products, including surfactants, paper protectants, packaging materials, textile items, carpets, cleaning products, nonstick coatings, and firefighting foams [10,11,12]. Common sources of PFAS exposure include food, contaminated water, household products, and dust [10]. PFAS can activate peroxisome proliferator-activated receptors (PPARs), which play important roles in adipogenesis [13,14]. A growing body of epidemiological research suggests that certain PFAS might be associated with obesity among children, adolescents, and non-pregnant adults [15]. However, less is known regarding the possible metabolic effects of PFAS among pregnant individuals. Maternal weight and metabolism change substantially during pregnancy and following delivery, which may indicate that pregnancy is a highly sensitive time window for PFAS exposure [16]. However, only one study has investigated the association of PFAS with postpartum weight retention; this study reported that higher plasma concentrations of PFAS as a mixture during pregnancy were suggestively associated with greater postpartum weight retention [17]. In particular, perfluorooctanoate (PFOA) was associated with a greater 1-year postpartum weight retention, and both PFOA and perfluorooctane sulfonate (PFOS) were associated with a higher 3-year postpartum weight retention [17]. These associations were stronger among individuals with a higher pre-pregnancy BMI [17]. However, participants in this study were enrolled in 1999–2002, before the phaseout of long-alkyl chain PFAS such as PFOS and PFOA, and had plasma PFAS concentrations considerably higher than current concentrations in the U.S. general population [10,17,18]. Thus, additional research conducted among pregnant individuals with contemporary PFAS exposure profiles is of interest.

Our study aimed to examine the association between PFAS mixture plasma concentrations during pregnancy and postpartum weight retention using a prospective cohort of pregnant individuals from rural areas. We further aimed to assess whether pre-pregnancy BMI and GWG modify the association between PFAS mixture concentrations and postpartum weight retention.

## 2. Materials and Methods

### 2.1. Study Population

The New Hampshire Birth Cohort Study (NHBCS) is an ongoing prospective cohort study launched in 2009. Pregnant individuals seeking prenatal care in New Hampshire were enrolled during their prenatal care visits if they met the following eligibility criteria: 18–45 years old, English speaking and literate, consuming drinking water from a private well or other unregulated water supply system at home, planning to stay in the same residence during pregnancy, and single gestation. Participants who were enrolled between 2009 and 2015 were recruited at approximately 24–28 gestational weeks, and participants enrolled between 2016 and 2018 were recruited at ~12 gestational weeks. The current study used data from a subset of 517 participants who had available plasma PFAS concentration quantified in November 2018–February 2019 and completed a maternal postpartum cardiometabolic health survey in 2020. The study was approved by the Committee for the Protection of Human Subjects at Dartmouth College. Written informed consent was obtained from all participants.

### 2.2. Plasma PFAS during Pregnancy

Maternal blood samples were collected during pregnancy (mean = 28 weeks, standard deviation, SD = 2.4 weeks). Samples were processed into aliquots of plasma within 24 h after collection and stored at −80 °C before overnight shipment on dry ice to the Centers for Disease Control and Prevention (CDC) National Center for Environmental Health laboratory. On-line solid-phase-extraction liquid-chromatography tandem mass spectrometry was used to quantify plasma concentrations of PFAS, including perfluorohexane sul-onate (PFHxS), linear PFOS (n-PFOS), perfluoromethylheptane sulfonate isomers (Sm-PFOS), linear PFOA (n-PFOA), branched PFOA isomers (Sb-PFOA), perfluorononanoate (PFNA), perfluorodecanoate (PFDA), perfluoroundecanoate (PFUnDA), and 2-(N-methyl-perfluorooctane sulfonamido) acetate (MeFOSAA) [19]. PFOA and PFOS concentrations were calculated as the sum of linear and branched isomers (n-PFOA + Sb-PFOA; n-PFOS + Sm-PFOS). Each batch contained blanks and quality control samples for analysis. The limit of detection (LOD) for each PFAS was 0.1 ng/mL, and the LOD/√2 was used to impute values below the LOD [20]. Concentrations with a signal recorded to be >0.05 but <0.1 were assigned 0.1/√2. Concentrations with a signal recorded to be >0 and <0.05 were assigned 0.05/√2. PFUnDA and MeFOSAA were excluded from the main statistical analysis because their concentrations were below the LOD for more than 60% of participants. In sensitivity analyses, we created indicator variables for PFUnDA and MeFOSAA (detectable vs. non-detectable) and included them in the mixture analysis. The CDC laboratory’s involvement did not constitute engagement in human-subject research.

### 2.3. Weight Retention

Participants completed a maternal postpartum cardiometabolic health survey online in 2020. Given the variability of enrollment year (2009–2018), these surveys were completed on average 7 years (range: 2–11) after the study pregnancy. Participants self-reported their current weight and updated information about lifestyle factors, pregnancy history, and health conditions after the study pregnancy. Pre-pregnancy weight and height were obtained from medical records or prenatal questionnaires if medical records were not available. We calculated postpartum weight change as the difference between self-reported weight (kg) from the cardiometabolic health survey and pre-pregnancy weight (kg). Of the 517 participants with available PFAS and cardiometabolic health survey data, we excluded participants with missing self-reported postpartum weight (n = 18), participants who were pregnant at the time of the cardiometabolic health survey (n = 16), and participants with missing pre-pregnancy weight (n = 1).

### 2.4. Covariates

Participants completed a prenatal baseline questionnaire and a food frequency questionnaire (FFQ) at enrollment, which collected comprehensive information on demographic, lifestyle, and dietary factors, as well as medical history and previous pregnancy history. At 2 weeks postpartum, participants were asked to complete a postpartum questionnaire including updated medical history, health conditions, and lifestyle factors during pregnancy. Medical records were used to obtain medical history and health conditions during the study pregnancy and at delivery. To account for diet quality during pregnancy, we constructed the Alternative Healthy Eating Index-2010 (AHEI-2010, range 0–110) using data from the FFQ. A higher AHEI-2010 score indicates a lower risk of future chronic diseases among Western populations [21,22]. Weight measures during pregnancy were abstracted from medical records. GWG was calculated as the difference between the weight at the last prenatal visit or delivery and pre-pregnancy weight (kg). We calculated the GWG Z-score specific to gestational age and pre-pregnancy BMI groups for each participant [23].

### 2.5. Statistical Analysis

#### 2.5.1. Main Analyses

All statistical analyses were conducted using R 4.0.2. We used Spearman correlation coefficients to examine the pairwise correlations between PFAS. Maternal plasma PFAS concentrations were log2-transformed to diminish the effect of extreme values.

Bayesian kernel machine regression (BKMR) was used to examine the association between the PFAS mixture and postpartum weight change. BKMR is a flexible statistical method that can assess the overall effect of the PFAS mixture on postpartum weight change and capture potential non-linear associations between PFAS and postpartum weight change as well as interactions between individual PFAS, while accounting for high correlations between individual PFAS and adjusting for confounders. Our main BKMR model was expressed as $Y_{i}=h\left(PFHxS_{i},\;PFOS_{i},\;PFOA_{i},\;PFNA_{i},\;PFDA_{i}\right)+\beta Z_{i}+\epsilon_{i}$, where ***Y*** represents the continuous postpartum weight change (kg); ***h*** () is a kernel function that can flexibly model the potential non-linear, non-additive effects of PFAS; ***PFHxS***, ***PFOS***, ***PFOA***, ***PFNA***, and ***PFDA*** are log2-transformed and then centered and scaled by the corresponding mean and SD; and ***Z*** represents confounders. The continuous confounders were centered and scaled by the corresponding mean and SD prior to fitting BKMR models. We conducted our analyses using the “bkmr” package in R 4.0.2. We used a Gaussian kernel with default priors and 100,000 Markov chain Monte Carlo iterations. Posterior inclusion probabilities (PIPs) were used to determine the importance of individual PFAS for postpartum weight change.

We selected the following covariates as potential confounders based on a priori knowledge: maternal age at enrollment, maternal highest education level, self-reported race/ethnicity (as a proxy for structural inequality and racism), marital status, pre-pregnancy BMI, smoking status, parity, previous lactation duration, diet quality during pregnancy, and physical activity before pregnancy. To account for the impact of plasma volume expansion and placental transfer on PFAS concentrations, we also adjusted for the gestational week of blood sample collection [24]. Additionally, participants who were enrolled in earlier calendar years had relatively higher plasma PFAS concentrations. They also had a longer follow-up time to when postpartum weight was collected. Given the variability of participants’ follow-up time (2–11 postpartum years since study pregnancy), we further adjusted for enrollment year and additional pregnancies after the study pregnancy. All models were adjusted for the following covariates: maternal age at enrollment (continuous, years), maternal highest education level (completed college, did not complete college), race/ethnicity (non-Hispanic White persons, persons with other race/ethnicity), marital status (married, other), pre-pregnancy BMI (continuous, kg/m^2^), self-reported smoking status (yes, no), self-reported second-hand tobacco smoke exposure during pregnancy (yes, no), parity (0, ≥1), previous lactation duration before the study pregnancy (continuous, months), AHEI-2010 score during pregnancy (continuous), sedentary time before the study pregnancy (continuous, hours/week), vigorous exercise time before the study pregnancy (continuous, hours/week), gestational week of blood sample collection (continuous, weeks), enrollment year (continuous, year), and indicator of having additional pregnancies following study pregnancy (yes, no).

We also used multivariable linear regression models for individual PFAS to quantify the effects of PFAS concentrations during pregnancy on maternal postpartum weight change, adjusting for confounders listed above.

#### 2.5.2. Secondary Analyses

Pre-pregnancy BMI may be an effect modifier of the association between prenatal PFAS and postpartum weight change [17]. We included pre-pregnancy BMI in the kernel function with the PFAS to assess potential interactions between them. We also stratified our analyses based on the pre-pregnancy BMI categories (healthy/underweight BMI < 25 kg/m^2^, overweight/obese BMI ≥ 25 kg/m^2^) and built BKMR models for each stratum. In the linear regression models, we included interaction terms between individual PFAS and binary pre-pregnancy BMI (healthy/underweight vs. overweight/obese) to evaluate potential effect modification by pre-pregnancy BMI.

To assess whether GWG is a potential effect modifier, we included the GWG Z-score in the kernel function with PFAS to detect potential interactions between them. We further built BKMR models for two pre-pregnancy BMI strata with PFAS and the GWG Z-score in the kernel function. In the linear regression models, we included interaction terms between individual PFAS and the GWG Z-score to evaluate effect modification by GWG.

#### 2.5.3. Sensitivity Analyses

To assess the impact of having additional pregnancies after the study pregnancy, we included interaction terms between individual PFAS and the indicator of having additional pregnancies after the study pregnancy (yes vs. no) in the linear regression models.

All BKMR and linear regression models included 433 participants with complete covariate information. However, in sensitivity analyses, we also conducted multiple imputation using the “mice” package in R 4.0.2. We generated 50 imputed datasets for 482 participants with available exposure and outcome data. We randomly selected one imputed dataset for BKMR analysis. The linear regression results were pooled from 50 imputed datasets.

In a sensitivity analysis, we also included binary PFUnDA and MeFOSAA (detectable vs. non-detectable) together with continuous PFHxS, PFOS, PFOA, PFNA, and PFDA in the kernel for the mixture analysis.

## 3. Results

**Figure 1:**
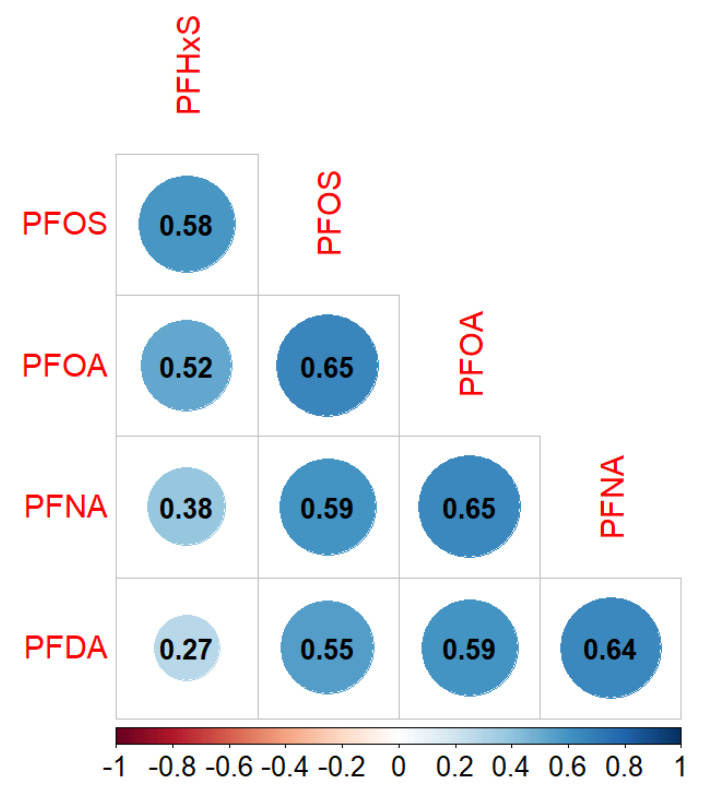
Spearman correlation coefficient estimates between plasma PFAS concentrations during pregnancy in the New Hampshire Birth Cohort Study (NHBCS) (n = 482).

The majority (75%) of the 482 participants with available PFAS and postpartum weight change data were enrolled between 2012 and 2015 with postpartum weight information collected 5–8 years after the study pregnancy (Table 1). On average, participants were 32 years old at the time of enrollment (SD = 4.6). Most participants were non-Hispanic White persons (93.6%), married (84.2%), and had a college degree or above (72.4%) (Table 1). The mean pre-pregnancy BMI was 26 kg/m^2^ (SD = 6.0), and the mean GWG was 15 kg (SD = 6.7) (Table 1). Participants had gained 4.1 kg (SD = 8.8) on average at the time of completing the cardiometabolic health survey in 2020 compared to pre-pregnancy (Table 1). Approximately 31.5% participants had additional pregnancies following the study pregnancy. On average, these participants (n = 152) had one additional pregnancy after the study pregnancy (SD = 0.65). Median PFAS concentrations in maternal plasma were 0.7 ng/mL (IQR = 0.50–1.00) for PFHxS, 3.2 ng/mL (IQR = 2.22–4.70) for PFOS, 1.1 ng/mL (IQR = 0.74–1.64) for PFOA, 0.5 ng/mL (IQR = 0.32–0.70) for PFNA, and 0.1 ng/mL (IQR =< LOD–0.20) for PFDA (Table 1). Spearman correlation coefficient estimates between the five PFAS ranged from 0.27 to 0.65, with most PFAS moderately and positively correlated with each other (Figure 1).

Compared to those with available PFAS data but missing postpartum weight change data (n = 454), participants included in the analysis (n = 482) had comparable plasma PFAS concentrations; however, multiparous participants (parity ≥ 1) were slightly over-represented in the analytic population (Table S1). Compared to those with available postpartum weight change data but missing PFAS data (n = 339), participants included in the analysis (n = 482) had similar weight change after the study pregnancy (Table S1). Overall, the distributions of demographic characteristics were relatively comparable between participants included in the analysis and those who were excluded (Table S1).

### 3.1. PFAS Mixture and Postpartum Weight Change

**Figure 2:**
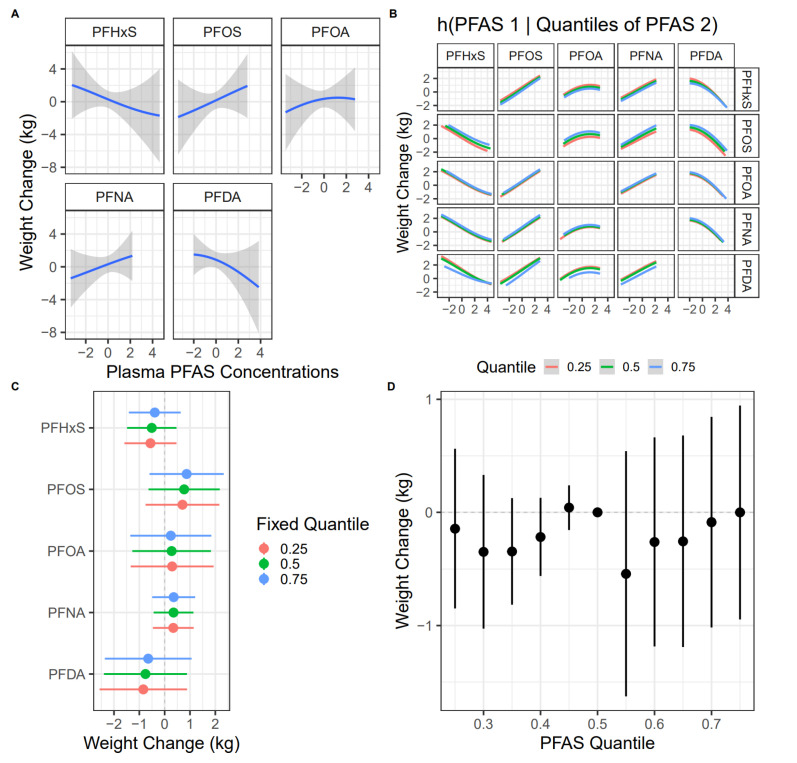
Associations between PFAS mixture plasma concentrations during pregnancy and postpartum weight change (kg) in the New Hampshire Birth Cohort Study (NHBCS) (n = 433). (**A**): Associations between individual PFAS and postpartum weight change when other PFAS are fixed at their median values. (**B**): Associations between individual PFAS and postpartum weight change when setting a second PFAS to its 25th, 50th, and 75th percentile values, holding the rest of the PFAS at their median values. (**C**): Associations between an individual PFAS and postpartum weight change when setting other PFAS simultaneously to their 25th, 50th, and 75th percentile values. (**D**): Difference in postpartum weight change when simultaneously setting all PFAS to different deciles compared with their median values. PFAS concentrations were log2-transformed and centered by their means and scaled by their standard deviations.

PFOS, PFOA, and PFNA had relatively higher PIPs compared to PFHxS and PFDA. Higher concentrations of PFOS, PFOA, and PFNA in maternal plasma were associated with greater postpartum weight change, while higher concentrations of PFHxS and PFDA were associated with a smaller postpartum weight change (Figure 2A). An interquartile range increase in plasma PFOS was associated with a 0.77 (95% credible interval: −0.64, 2.18) kg greater postpartum weight change, setting other PFAS to their median values. BKMR models did not show strong evidence of interactions between the different PFAS, and the associations between an individual PFAS and postpartum weight change were approximately linear (Figure 2B,C). The overall association between the PFAS mixture and postpartum weight change was close to null (Figure 2D).

### 3.2. Individual PFAS and Postpartum Weight Change

Given that BKMR models did not detect obvious non-linear or non-additive associations between the PFAS and postpartum weight change (Figure 2A,B), we further used multivariable linear regression models to quantify the associations for individual PFAS. The directionality of the associations between individual PFAS and postpartum weight change from linear regression models was consistent with the BKMR model. Each doubling of plasma PFOS, PFOA, and PFNA concentrations was associated with 0.80 kg (95% CI: −0.28, 1.88), 0.57 kg (95% CI: −0.70, 1.84), and 0.54 kg (95% CI: −0.26, 1.34) greater postpartum weight change, respectively, adjusting for relevant covariates (Table 2). Associations for PFHxS and PFDA were close to null (0.02 kg, 95% CI: −0.81, 0.86; −0.01 kg, 95% CI: −0.93, 0.92, respectively) (Table 2).

### 3.3. Effect Modification by Pre-Pregnancy BMI

BKMR models suggested potential interactions between maternal pre-pregnancy BMI and PFAS (Figure S1). The associations for PFAS, especially PFOS, may vary by pre-pregnancy BMI (Figures S1 and S2). Among individuals who had a healthy/underweight BMI prior to pregnancy, an interquartile range increase in plasma PFOS concentrations was associated with a −0.07 (95% credible interval: −1.28, 1.14) kg postpartum weight change, setting other PFAS to their median values. However, among individuals who had an obese/overweight BMI prior to pregnancy, an interquartile range increase in plasma PFOS concentrations was associated with a 1.81 (95% credible interval: −0.88, 4.50) kg greater postpartum weight, setting other PFAS to their median values. In the linear regression models, pre-pregnancy BMI was a statistically significant effect modifier of the association between PFOS and postpartum weight change (*p*-value for interaction = 0.06). Each doubling of PFOS maternal plasma concentrations was associated with 1.76 kg (95% CI: 0.31, 3.22) greater postpartum weight change among participants who had an obese/overweight BMI before the study pregnancy, whereas this association was null among participants who had healthy/underweight BMI before the study pregnancy (0.10 kg, 95% CI: −1.24, 1.44) (Table 2). The associations for PFOA and PFNA were also stronger among participants who had an obese/overweight BMI before the study pregnancy compared to those who had healthy/underweight BMI, although the differences were not statistically significant (Table 2).

### 3.4. Effect Modification by GWG

BKMR models did not show strong evidence for interactions between GWG Z-score and any of the PFAS (Figure S3). Consistent with the BKMR findings, cross-product terms for PFAS and the GWG Z-score were not statistically significant in the linear regression models.

### 3.5. Sensitivity Analyses

PFAS associations with postpartum weight retention from BKMR models were similar for participants who had additional pregnancies after the study pregnancy compared with participants who did not (Figure S4). In linear regression models, the association between PFNA and postpartum weight retention appeared stronger among participants who had additional pregnancies after the study pregnancy (Table S2). Plasma PFNA concentrations were relatively higher among participants who had additional pregnancies after the study pregnancy (median: 0.6, IQR: 0.4–0.8) compared to participants who did not (median: 0.5, IQR: 0.3–0.7), which may partially explain the observed difference.

Results were similar when using multiple imputation compared to complete case analyses (Table S3, Figure S5). The directionality of associations between PFHxS, PFOS, PFOA, PFNA, and PFDA and postpartum weight change remained the same when binary PFUnDA and MeFOSAA (detectable vs. non-detectable) were added to the kernel function.

## 4. Discussion

In our study of mother–child pairs in northern rural New England, higher PFOS concentrations in maternal plasma during pregnancy were associated with greater weight retention after pregnancy, especially among participants who had a higher BMI prior to the study pregnancy. To our knowledge, this is the first study to assess the long-term effects of PFAS mixture plasma concentrations during pregnancy on postpartum weight change (7 years after the study pregnancy on average).

Our findings are consistent with a previous study, which examined relationships between PFAS concentrations in maternal plasma during pregnancy and postpartum weight change over a shorter follow-up period. Mitro et al. examined the effects of PFAS plasma concentrations both individually and as a mixture during pregnancy on postpartum weight retention among participants enrolled during 1999–2002 in Project Viva, a prospective cohort in eastern Massachusetts, USA [17]. They found that plasma PFOA and PFOS concentrations were positively associated with 3-year postpartum weight change [17]. Similar to our findings, these associations were stronger among participants with a higher pre-pregnancy BMI [17]. Given that most of the participants in our study were 5–8 years postpartum when postpartum weight was collected, our study suggests that prenatal PFAS exposure might also have long-term effects on postpartum weight change. Although we observed a similar positive association between PFOA and postpartum weight retention, this was not statistically significant, which may relate to the smaller sample size or relatively lower concentrations of plasma PFAS in the NHBCS compared to Project Viva. Unlike Project Viva, participants in the NHBCS were enrolled starting in 2009, after the phaseout of several long-alkyl chain PFAS including PFOA and PFOS. Serum concentrations of PFOA and PFOS have steadily decreased in the general U.S. population from 1999 to 2018 based on the National Health and Nutrition Examination Survey (NHANES) [17,18]. Nevertheless, our study suggests that the adverse effects of these PFAS remain a concern because of the environmental persistence and long biological half-lives of long-alkyl chain PFAS.

Several studies have investigated the association between PFAS exposure and GWG [17,25,26,27,28]. Two studies found higher PFOS concentrations were related to higher GWG measures among individuals with a pre-pregnancy BMI < 25 kg/m^2^ [25,26]. One study found a positive association between PFNA and GWG among individuals with a pre-pregnancy BMI < 25 kg/m^2^, while the other found a weak positive association between PFNA and GWG among individuals with a pre-pregnancy BMI ≥ 25 kg/m^2^ [27,28]. Although results have been inconsistent, these findings indicate that pre-pregnancy BMI may modify associations between PFAS and maternal weight change. In support of this, we found that the positive association between plasma PFOS concentrations and postpartum weight change in the NHBCS was stronger among participants with a higher pre-pregnancy BMI. We further assessed potential effect modification by GWG during the study pregnancy, creating a GWG Z-score to account for the influence of pre-pregnancy BMI and gestational age on GWG. However, associations between plasma PFAS concentrations and postpartum weight change did not vary by GWG Z-score. Collectively, these findings suggest that individuals with a higher pre-pregnancy BMI may be particularly susceptible to the adverse effects of PFAS exposure on postpartum weight change.

Findings from other studies have suggested potential links between PFAS exposure and weight change among non-pregnant women. In the POUNDS (Preventing Overweight Using Novel Dietary Strategies) Lost trial, Liu et al. found that plasma PFAS concentrations at baseline were positively associated with more weight regain during the 6–24 months after weight loss during the first 6 months, especially among women [29]. They also found that higher plasma PFOS and PFNA concentrations at baseline were related to a larger decrease in resting metabolic rate during the first 6 months of the weight-loss period and a smaller increase in resting metabolic rate during the 6–24 months of weight regain period [29]. Likewise, in the Diabetes Prevention Program Outcomes Study, Cardenas et al. found that higher plasma PFAS concentrations were associated with greater weight gain among participants in the placebo group (no intervention of exercise and diet) [30]. Collectively, the studies among both pregnant and non-pregnant individuals suggest that PFAS may adversely influence weight retention and weight gain among adults.

There are several possible mechanisms that may explain the associations between PFAS and weight change. Endocrine-disrupting chemicals can modify metabolic processes and affect weight gain through nuclear receptor activation, disruption of epigenetic regulation, and interference with hormone regulation, among other potential mechanisms [31]. The potential modes of action for PFAS on weight gain and retention may include interfering with multiple nuclear receptors [31]. PFAS can activate PPARα and PPARγ, which play essential roles in adipocyte differentiation, lipid metabolism, and fatty acid oxidation [32,33]. In vivo studies have demonstrated that the absence of PPARγ in adipose tissues can hamper adipocyte differentiation and cause lipodystrophy [34,35]. Partial lipodystrophy had also been observed in clinic patients with a heterozygous PPARγ mutation [36]. Apart from activation of PPARγ, PFAS may promote adipogenesis through other regulators. Liu et al. found that PFOS could increase mRNA expression of PPARγ, CCAAT/enhancer-binding protein-α (C/EBPα), lipoprotein lipase, and leptin [37]. Furthermore, animal and in vitro studies have shown that certain PFAS, including PFOA and PFOS, have estrogenic activity and directly interact with estrogen receptors (ERs) [38,39,40,41,42,43]. ERs play an important part in energy balance, glucose homeostasis, and lipid metabolism [44,45]. Research has indicated that PFAS may disturb transcription mediated by ERs and interfere with ER signaling pathways [43].

Our study has several limitations. We could not rule out the possibility of social desirability bias because we used self-reported postpartum weight from a survey. However, while participants may underreport their postpartum weight, this measurement error is unlikely to be related to their PFAS plasma concentrations. Thus, the true associations between PFAS exposure and postpartum weight retention may be stronger than those observed in our study. We only had a single measure of postpartum weight after the study pregnancy. Additional research would increase the understanding of the effects of PFAS exposure on weight change trajectories after pregnancy using longitudinal measurements of postpartum weight. This would help identify risk factors for high rates of weight retention after pregnancy and the timing of effective interventions to prevent weight retention. The analysis was limited to 482 participants in the NHBCS due to data availability. More than half (n = 482, 51.5%) of participants with plasma PFAS measurements from pregnancy (n = 936) also completed the maternal postpartum cardiometabolic health survey. To evaluate potential selection bias, we compared the distributions of PFAS concentrations, outcome, and covariates between the analytic population and participants who were excluded from the analysis. Although we did not find substantial differences (Table S1), we cannot entirely rule out the possibility of selection bias due to potential differences in unmeasured covariates. In addition, 78.6% of participants who had obese/overweight BMI prior to the study pregnancy were categorized as having excessive gestational weight gain based on the Institute of Medicine guidelines, which provides ranges of recommended GWG given an individual’s pre-pregnancy BMI [46]. Therefore, we had limited power to detect effect modification by GWG categories (inadequate/healthy gestational weight gain; excessive gestational weight gain). Participants in the NHBCS were enrolled from rural populations that have been historically understudied [47]. They mostly rely on private wells for drinking water and therefore have a high risk of being exposed to environmental drinking water contaminants such as arsenic. Our study can therefore inform interventions to improve overall health of these vulnerable populations. However, it is also important to note that our findings may not be generalizable to urban populations. The majority of participants in the NHBCS are non-Hispanic White persons, reflective of the demographics of New Hampshire. Because of our initial inclusion criteria of having a private water system supplying their residence, the socioeconomic status of our participants tended to be representative of homeowners and non-smokers. Plasma PFAS concentrations in the NHBCS are also somewhat lower than the U.S. general population based on NHANES data during the same time period [18]. This finding may reflect lower PFAS exposures in our study population compared to NHANES or could relate to the timing of blood collection in the NHBCS, as samples were collected at ~28 weeks gestation, and plasma PFAS concentrations tend to decrease during pregnancy because of plasma volume expansion and placenta transfer [24,48,49,50].

Our study also had many strengths. To our knowledge, this was the first to evaluate the long-term effects of a PFAS mixture on postpartum weight retention among pregnant individuals in a contemporary U.S. cohort. We had comprehensive data collected from medical records and questionnaires in a large prospective cohort study, which allowed us to adjust for key covariates including demographic, reproductive, dietary, and physical activity factors. Additionally, BKMR models allowed us to assess potential non-linear, non-additive effects of PFAS plasma concentrations during pregnancy, while accounting for correlations between these contaminants.

## 5. Conclusions

In conclusion, our results indicate that higher plasma PFOS concentrations during pregnancy may be associated with greater long-term weight retention after pregnancy. They further suggest that individuals with a higher pre-pregnancy BMI may be particularly vulnerable. Our findings suggest that targeted interventions to reduce PFAS exposure among susceptible populations may play a partial role in reducing the obesity epidemic and improve cardiometabolic health for mothers and offspring.

## Figures and Tables

**Table 1:** Distribution of participants’ characteristics and plasma PFAS concentrations in the New Hampshire Birth Cohort Study (n = 482).

Characteristics	Mean ± SD/N (%)	
**Maternal Age (years)**	32 ± 4.6	
**Self-Reported Race/Ethnicity**		
Non-Hispanic White	451 (93.6)	
Other	30 (6.2)	
Missing	1 (0.2)	
**Maternal Highest Education**		
Less Than College	110 (22.8)	
College Graduate or Above	349 (72.4)	
Missing	23 (4.8)	
**Marital Status**		
Married	406 (84.2)	
Other	55 (11.4)	
Missing	21 (4.4)	
**Maternal Pre-Pregnancy BMI (kg/m^2^)**	26 ± 6.0	
**Parity**		
0	186 (38.6)	
≥1	293 (60.8)	
Missing	3 (0.6)	
**Lactation Duration Before the Study Pregnancy (months)**	9 ± 12.4	
**Weight Gain During Pregnancy (kg)**	15 ± 6.7	
**Smoking Status**		
Never	427 (88.6)	
Current	27 (5.6)	
Former	28 (5.8)	
**Alternative Healthy Eating Index (AHEI 2010) Score**	58 ± 12.7	
**Physical Activity**		
Sedentary Time (hours/week)	8.5 ± 6.0	
Vigorous Exercise Time (hours/week)	3.0 ± 3.4	
**Gestational Week of Blood Sample (weeks)**	28 ± 2.4	
**Additional Pregnancies After the Study Pregnancy**		
Yes	152 (31.5)	
No	328 (68.0)	
Missing	2 (0.4)	
**Postpartum Weight Change (kg)**	4.1 ± 8.8	
		**Median (IQR)**
**Plasma PFAS Concentrations (ng/mL)**		
PFHxS		0.7 (0.50, 1.00)
PFOS		3.2 (2.22, 4.70)
PFOA		1.1 (0.74, 1.64)
PFNA		0.5 (0.32, 0.70)
PFDA		0.1 (<LOD ^1^, 0.20)
		**Median (Range)**
**Enrollment Year**		
		2013 (2009–2018)

^1^ LOD (limit of detection) for PFAS in plasma: 0.1 (ng/mL). SD: standard deviation; IQR: interquartile range.

**Table 2:** Associations between individual plasma PFAS concentrations during pregnancy (2009–2018) and postpartum weight change (kg) in the New Hampshire Birth Cohort Study (n = 433).

PFAS	Postpartum Weight Change (95% CI)		
	Overall (n = 433)	Obese/Overweight (n = 191)	Healthy/Underweight (n = 242)
PFHxS	0.02 (−0.81, 0.86)	0.15 (−0.98, 1.27)	0.05 (−1.09, 1.19)
PFOS	0.80 (−0.28, 1.88)	1.76 (0.31, 3.22)	0.10 (−1.24, 1.44)
PFOA	0.57 (−0.70, 1.84)	1.39 (−0.27, 3.04)	0.17 (−1.31, 1.65)
PFNA	0.54 (−0.26, 1.34)	1.04 (−0.19, 2.28)	0.22 (−0.74, 1.18)
PFDA	−0.01 (−0.93, 0.92)	0.49 (−0.84, 1.81)	−0.40 (−1.59, 0.78)

Notes: All models were adjusted for maternal age at enrollment (continuous, years), ma-ternal highest education level (≥college, <college), self-reported race/ethnicity (non-Hispanic White persons, persons with other race/ethnicity), marital status (mar-ried, other), pre-pregnancy BMI (continuous, kg/m2), self-reported smoking status (yes, no), self-reported second-hand tobacco smoke exposure during pregnancy (yes, no), parity (0, ≥1), previous lactation duration before the study pregnancy (continuous, months), AHEI-2010 score during pregnancy (continuous), sedentary time before the study pregnancy (continuous, hours/week), vigorous exercise time before the study pregnancy (continuous, hours/week), gestational week of blood sample at collection (continuous, weeks), enrollment year (continuous, year), and indicator of having addi-tional pregnancies following the study pregnancy (yes, no).; CI: confidence interval.

## Data Availability

Data will be made available to outside entities upon reasonable request and in accordance with the Dartmouth CPHS and the Dartmouth College IRB by contacting the NHBCS director, Dr. Margaret Karagas (margaret.r.karagas@dartmouth.edu).

## Supplementary Materials

The following supporting information can be downloaded at: https://www.mdpi.com/article/10.3390/toxics11050450/s1, Table S1: Comparison between New Hampshire Birth Cohort Study participants included in the analysis (n = 482) and participants excluded from the analysis (n = 793); Table S2: Associations between plasma PFAS concentrations during pregnancy and postpartum weight change (kg) based on indicator of having additional pregnancies after study pregnancy in the New Hampshire Birth Cohort Study (n = 433); Table S3: Pooled results from 50 imputed datasets regarding associations between individual plasma PFAS concentrations during pregnancy and postpartum weight change (kg) in the New Hampshire Birth Cohort Study (n = 482); Figure S1: Effect modification of pre-pregnancy body mass index (BMI) regarding associations between plasma PFAS concentrations during pregnancy and postpartum weight change (kg) in the New Hampshire Birth Cohort Study (NHBCS) (n = 433); Figure S2: Associations between PFAS mixture plasma concentrations during pregnancy and postpartum weight change (kg) stratified by pre-pregnancy BMI category (obese/overweight vs. healthy/underweight) in the New Hampshire Birth Cohort Study (NHBCS) (n = 433); Figure S3: Effect modification of gestational weight gain (GWG) Z-score regarding associations between plasma PFAS concentrations during pregnancy and postpartum weight change (kg) in the New Hampshire Birth Cohort Study (NHBCS) (n = 433); Figure S4: Associations between plasma PFAS concentrations during pregnancy and postpartum weight change (kg) based on an indicator of having additional pregnancies after the study pregnancy in the New Hampshire Birth Cohort Study (NHBCS) (n = 433); Figure S5: Associations between the PFAS mixture plasma concentrations during pregnancy and postpartum weight change (kg) in one randomly selected dataset from multiple imputation in the New Hampshire Birth Cohort Stud (NHBCS) (n = 482).

## Abbreviations

PFAS	Per- and polyfluoroalkyl substances
NHBCS	New Hampshire Birth Cohort Study
PFOS	Perfluorooctane sulfonate
PFOA	Perfluorooctanoate
PFNA	Perfluorononanoate
BMI	Body mass index
CVDs	Cardiovascular diseases
GWG	Gestational weight gain
PPAR	Peroxisome proliferator-activated receptor
SD	Standard deviation
CDC	Centers for Disease Control and Prevention
PFHxS	Perfluorohexane sulfonate
PFDA	Perfluorodecanoate
PFUnDA	Perfluoroundecanoate
MeFOSAA	2–(N–methyl–perfluorooctane sulfonamido) acetate
LOD	Limit of detection
FFQ	Food frequency questionnaire
AHEI-2010	Alternative Healthy Eating Index-2010
BKMR	Bayesian kernel machine regression
PIP	Posterior inclusion probability
IQR	Interquartile range
CI	Confidence interval
NHANES	National Health and Nutrition Examination Survey
POUNDS	Preventing Overweight Using Novel Dietary Strategies
C/EBPα	CCAAT/enhancer-binding protein-α
ER	Estrogen receptor
