# Supplementary material for: Per- and Polyfluoroalkyl Substances (PFAS) Mixture during Pregnancy and Postpartum Weight Retention in the New Hampshire Birth Cohort Study (NHBCS)
Source: Toxics. 2023 May 10;11(5):450. doi: 10.3390/toxics11050450 (PMC10223499; doi:10.3390/toxics11050450)
Supplement: Supplementary file 1 [file toxics-11-00450-s001.zip › toxics-2351741-supplementary.pdf]

toxics

MDPI

Supplementary Materials

Per- and Polyfluoroalkyl Substances (PFAS) Mixture During Pregnancy and Postpartum Weight Retention in the New Hampshire Birth Cohort Study (NHBCS)

Yuting Wang, Caitlin Howe, Lisa G. Gallagher, Julianne Cook Botelho, Antonia M. Calafat, Margaret R. Karagas and Megan E. Romano

S1

Table S1. Comparison between New Hampshire Birth Cohort Study participants included in the analysis (n = 482) and participants excluded from the analysis (n = 793).

S2

Table S2. Associations between plasma PFAS concentrations during pregnancy and postpartum weight change (kg) based on indicator of having additional pregnancies after study pregnancy in the New Hampshire Birth Cohort Study (n = 433).

S3

Table S3. Pooled results from 50 imputed datasets regarding associations between individual plasma PFAS concentrations during pregnancy and postpartum weight change (kg) in the New Hampshire Birth Cohort Study (n = 482).

S4

Figure S1. Effect modification of pre-pregnancy body mass index (BMI) regarding associations between plasma PFAS concentrations during pregnancy and postpartum weight change (kg) in the New Hampshire Birth Cohort Study (NHBCS) (n = 433).

S5

Figure S2. Associations between PFAS mixture plasma concentrations during pregnancy and postpartum weight change (kg) stratified by pre-pregnancy BMI category (obese/overweight vs. healthy/underweight) in the New Hampshire Birth Cohort Study (NHBCS) (n = 433).

S6

Figure S3. Effect modification of gestational weight gain (GWG) Z-score regarding associations between plasma PFAS concentrations during pregnancy and postpartum weight change (kg) in the New Hampshire Birth Cohort Study (NHBCS) (n = 433).

S7

Figure S4. Associations between plasma PFAS concentrations during pregnancy and postpartum weight change (kg) based on an indicator of having additional pregnancies after the study pregnancy in the New Hampshire Birth Cohort Study (NHBCS) (n = 433).

S8

Figure S5. Associations between the PFAS mixture plasma concentrations during pregnancy and postpartum weight change (kg) in one randomly selected dataset from multiple imputation in the New Hampshire Birth Cohort Study (NHBCS) (n = 482).

Table S1. Comparison between New Hampshire Birth Cohort Study participants included in the analysis (n = 482) and participants excluded from the analysis (n = 793).

Characteristics	Participants with plasma PFAS concentrations and postpartum weight change (N = 482)	Participants with plasma PFAS concentrations and missing postpartum weight change (N = 454)	Participants with postpartum weight change and missing plasma PFAS concentrations (N = 339)
	Mean ± SD / N (%)		
**Maternal Age (years)**	32 ± 4.6	31 ± 4.7	31 ± 4.8
**Self-reported Race/Ethnicity**			
Non-Hispanic White	451 (93.6)	418 (92.1)	302 (89.1)
Other	30 (6.2)	27 (5.9)	22 (6.5)
Missing	1 (0.2)	9 (2.0)	15 (4.4)
**Maternal Highest Education**			
Less than college	110 (22.8)	133 (29.3)	92 (27.1)
College graduate or above	349 (72.4)	285 (62.8)	198 (58.4)
Missing	23 (4.8)	36 (7.9)	49 (14.5)
**Marital Status**			
Married	406 (84.2)	355 (78.2)	247 (72.9)
Other	55 (11.4)	63 (13.9)	44 (13.0)
Missing	21 (4.4)	36 (7.9)	48 (14.2)
**Maternal Pre-pregnancy BMI (kg/m^2)**	26 ± 6.0	26 ± 5.7	26 ± 5.6
**Parity**			
0	186 (38.6)	227 (50.0)	129 (38.1)
≥ 1	293 (60.8)	223 (49.1)	200 (59.0)
Missing	3 (0.6)	4 (0.9)	10 (2.9)
**Previous Lactation Duration (months)**	9 ± 12.4	7 ± 13.7	9 ± 17.0
**Weight Gain During Pregnancy (kg)**	15 ± 6.7	15 ± 6.4	15 ± 6.8
**Smoking Status**			
Never	427 (88.6)	356 (78.4)	289 (85.3)
Current	27 (5.6)	33 (7.3)	33 (9.7)
Former	28 (5.8)	28 (6.2)	17 (5.0)
Missing	0 (0)	37 (8.1)	0 (0)
**Alternative Healthy Eating Index-2010**	58 ± 12.7	56 ± 12.2	57 ± 12.7
**Physical Activity**			
Sedentary time (hours/week)	8.5 ± 6.0	9.5 ± 9.1	9.1 ± 6.8
Vigorous exercise time (hours/week)	3.0 ± 3.4	3.7 ± 6.2	3.0 ± 4.4
**Gestational Week of Blood Samples (weeks)**	28 ± 2.4	28 ± 2.4	-
**Addition Pregnancies After Study Pregnancy**			
Yes	152 (31.5)	-	128 (37.8)
No	328 (68.0)	-	211 (62.2)
Missing	2 (0.4)	-	0 (0)
**Postpartum Weight Change (kg)**	4.1 ± 8.8	-	4.6 ± 10.0
		Median (IQR)	
**Plasma PFAS Concentrations (ng/mL)**			
PFHxS	0.7 (0.50, 1.00)	0.8 (0.50, 1.10)	-
PFOS	3.2 (2.22, 4.70)	3.7 (2.40,5.50)	-
PFOA	1.1 (0.74, 1.64)	1.3 (0.84, 1.84)	-

PFNA	0.5 (0.32, 0.70)	0.5 (0.40, 0.80)	-
PFDA	0.1 (< LOD [1], 0.20)	0.2 (0.1, 0.20)	-
		**Median (Range)**	
**Enrollment Year**	2013 (2009-2018)	2012 (2009-2018)	2014 (2009-2018)

Notes: [1] LOD (limit of detection) for PFAS in plasma: 0.1 (ng/mL) SD: standard deviation; IQR: interquartile range.

Table S2. Associations between plasma PFAS concentrations during pregnancy and postpartum weight change (kg) based on indicator of having additional pregnancies after study pregnancy in the New Hampshire Birth Cohort Study (n = 433).

PFAS	Postpartum Weight Change (95% CI)	
	**With Additional Pregnancies (n=136)**	**Without Additional Pregnancies (n=297)**
PFHxS	-0.75 (-2.21, 0.72)	0.33 (-0.63, 1.30)
PFOS	1.31 (-0.36, 2.99)	0.55 (-0.69, 1.79)
PFOA	1.09 (-0.67, 2.85)	0.27 (-1.18, 1.72)
PFNA	1.46 (0.08, 2.85)	0.19 (-0.72, 1.01)
PFDA	0.68 (-0.87, 2.23)	-0.31 (-139, 0.77)

Notes: All models were adjusted for maternal age at enrollment (continuous, years), maternal highest education level (≥ college,< college), self-reported race/ethnicity (non-Hispanic White persons, persons with other race/ethnicity), marital status (married, other), pre-pregnancy BMI (continuous, kg/m^2), self-reported smoking status (yes, no), self-reported second-hand tobacco smoke exposure during pregnancy (yes, no), parity (0, ≥ 1), previous lactation duration before the study pregnancy (continuous, months), AHEI-2010 score during pregnancy (continuous), sedentary time before the study pregnancy (continuous, hours/week), vigorous exercise time before the study pregnancy (continuous, hours/week), gestational week of blood sample at collection (continuous, weeks), enrollment year (continuous, year); CI: confidence interval.

Table S3. Pooled results from 50 imputed datasets regarding associations between individual plasma PFAS concentrations during pregnancy and postpartum weight change (kg) in the New Hampshire Birth Cohort Study (n = 482).

PFAS	Postpartum Weight Change (95% CI)		
	**Overall (n=482)**	**Obese/Overweight (n=217)**	**Healthy/Underweight (n=265)**
PFHxS	0.06 (-0.75, 0.88)	0.19 (-0.90, 1.28)	0.05 (-1.07, 1.17)
PFOS	0.81 (-0.25, 1.87)	1.73 (0.32, 3.14)	0.11 (-1.20, 1.42)
PFOA	0.72 (-0.50, 1.95)	1.57 (-0.03, 3.16)	0.20 (-1.13, 1.72)
PFNA	0.69 (-0.08, 1.45)	1.09 (-0.09, 2.27)	0.43 (-0.50, 1.35)
PFDA	-0.01 (-0.90, 0.87)	0.10 (-1.17, 1.37)	-0.10 (-1.24, 1.04)

Notes: All models were adjusted for maternal age at enrollment (continuous, years), maternal highest education level (≥ college,< college), self-reported race/ethnicity (non-Hispanic White persons, persons with other race/ethnicity), marital status (married, other), pre-pregnancy BMI (continuous, kg/m^2), self-reported smoking status (yes, no), self-reported second-hand tobacco smoke exposure during pregnancy (yes, no), parity (0, ≥ 1), previous lactation duration before the study pregnancy (continuous, months), AHEI-2010 score during pregnancy (continuous), sedentary time before the study pregnancy (continuous, hours/week), vigorous exercise time before the study pregnancy (continuous, hours/week), gestational week of blood sample at collection (continuous, weeks), enrollment year (continuous, year), indicator of having additional pregnancies following the study pregnancy (yes, no); CI: confidence interval.

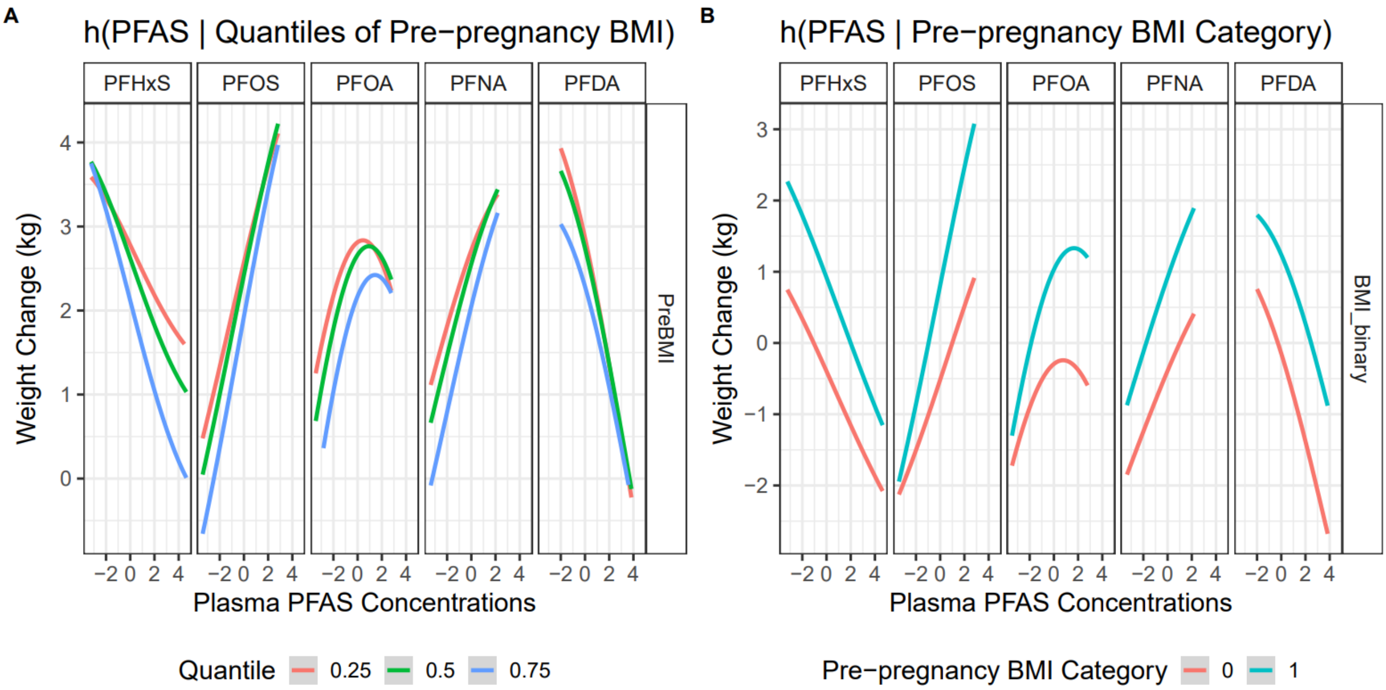

Figure S1. Effect modification of pre-pregnancy body mass index (BMI) regarding associations between plasma PFAS concentrations during pregnancy and postpartum weight change (kg) in the New Hampshire Birth Cohort Study (NHBCS) (n = 433). S1A: Associations between individual PFAS and postpartum weight change when pre-pregnancy BMI is fixed at its 25th, 50th, and 75th percentile values, and other PFAS are fixed at their median values. S1B: Associations between individual PFAS and postpartum weight change based on binary pre-pregnancy BMI (obese/overweight: blue) vs. (healthy/underweight: red). PFAS concentrations were log2-transformed and centered by their means and scaled by their standard deviations. Continuous pre-pregnancy BMI was centered by its mean and scaled by its standard deviation. Binary pre-pregnancy BMI categories: 0 (red)—healthy/underweight; 1 (blue)—obese/overweight.

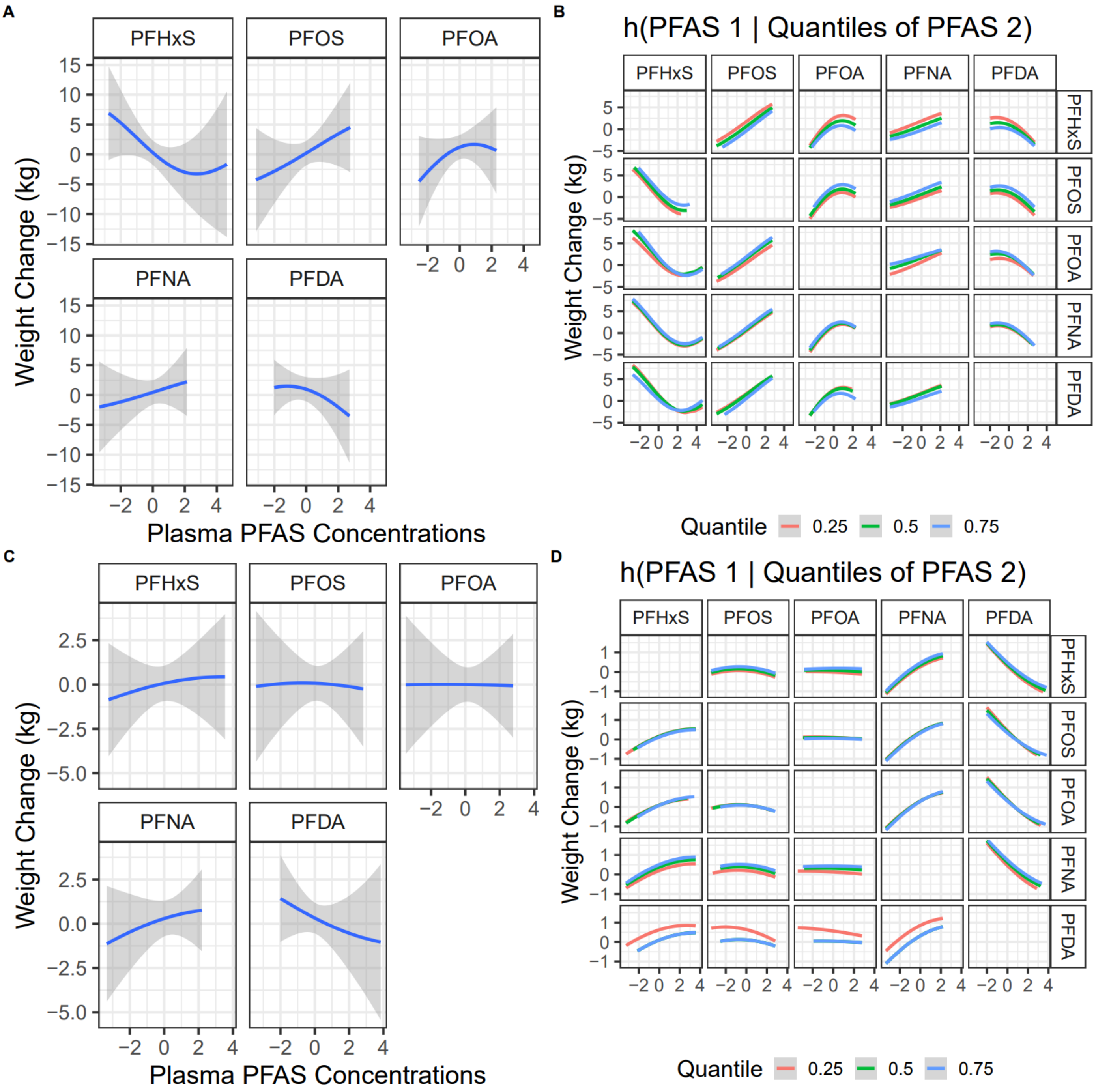

Figure S2. Associations between PFAS mixture plasma concentrations during pregnancy and postpartum weight change (kg) stratified by pre-pregnancy BMI category (obese/overweight vs. healthy/underweight) in the New Hampshire Birth Cohort Study (NHBCS) (n = 433). S2A: Associations between individual PFAS and postpartum weight change when other PFAS are fixed at their median values among obese/overweight participants. S2B: The association between individual PFAS and postpartum weight change when a second PFAS is fixed to its 25th, 50th, and 75th percentile values, and the rest of the PFAS are fixed at their median values among obese/overweight participants. S2C: Associations between individual PFAS and postpartum weight change when other PFAS are fixed at their median values among healthy/underweight participants. S2D: Associations between individual PFAS and postpartum weight change when a second PFAS is fixed at its 25th, 50th, and 75th percentile values, and the rest of the PFAS are fixed at their median values among healthy/underweight participants. PFAS concentrations were log2-transformed and centered by their means and scaled by their standard deviations.

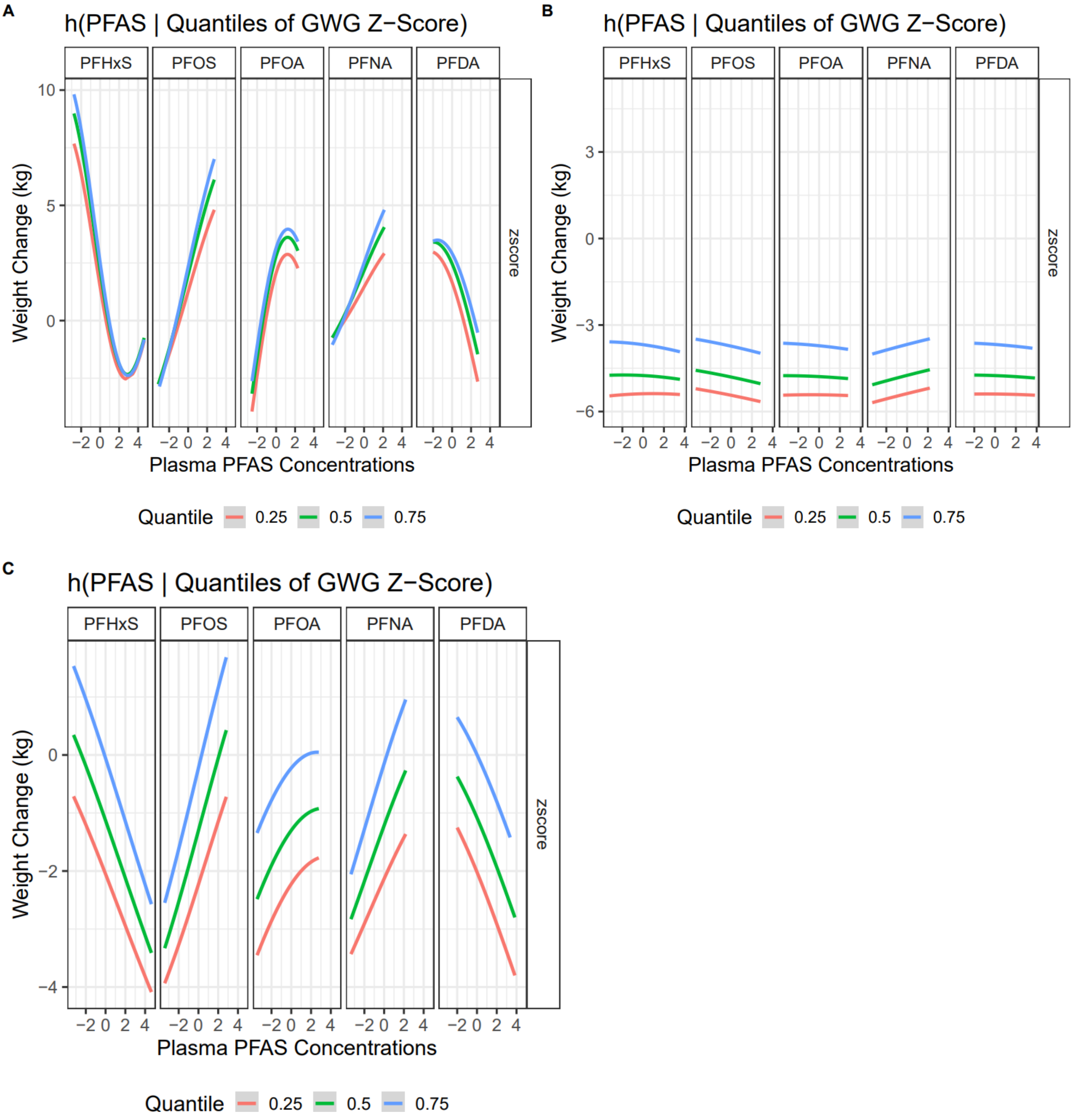

Figure S3. Effect modification of gestational weight gain (GWG) Z-score regarding associations between plasma PFAS concentrations during pregnancy and postpartum weight change (kg) in the New Hampshire Birth Cohort Study (NHBCS) (n = 433). S3A: Among participants who had obesity/overweight before the study pregnancy, associations between individual PFAS and postpartum weight change when the GWG Z-score is fixed at its 25th, 50th, and 75th percentile values, and other PFAS are fixed at their median values. S3B: Among participants who were healthy/underweight before the study pregnancy, associations between individual PFAS and postpartum weight change when the GWG Z-score is fixed at its 25th, 50th, and 75th percentile values, and other PFAS are fixed at their median values. S3C: Associations between individual PFAS and postpartum weight change when the GWG Z-score is fixed at its 25th, 50th, and 75th percentile values, and other PFAS are fixed at their median values. PFAS concentrations were log2-transformed and centered by their means and scaled by their standard deviations.

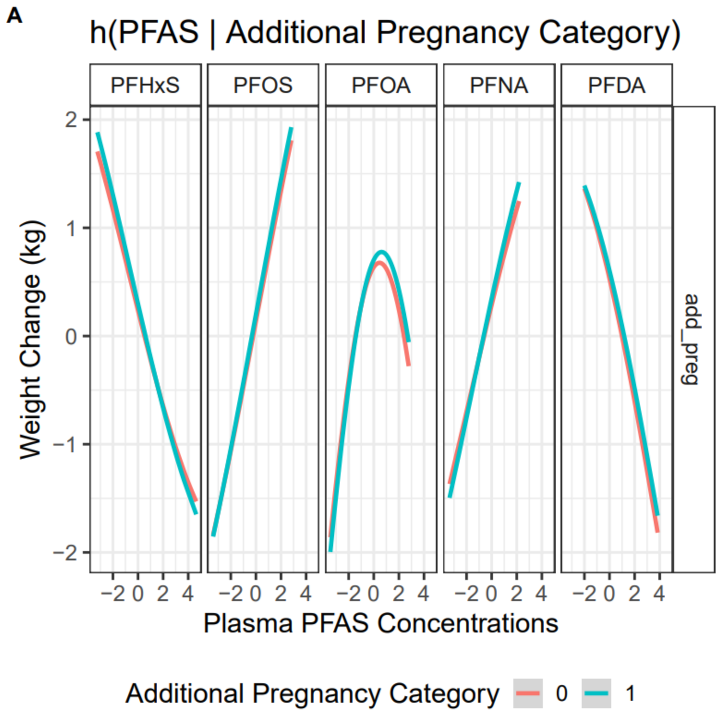

Figure S4. Associations between plasma PFAS concentrations during pregnancy and postpartum weight change (kg) based on an indicator of having additional pregnancies after the study pregnancy in the New Hampshire Birth Cohort Study (NHBCS) (n = 433). Associations between individual PFAS and postpartum weight change based on an indicator of having additional pregnancies after the study pregnancy (yes: 1 – blue) vs. (no: 0 – red). PFAS concentrations were log2-transformed and centered by their means and scaled by their standard deviations.

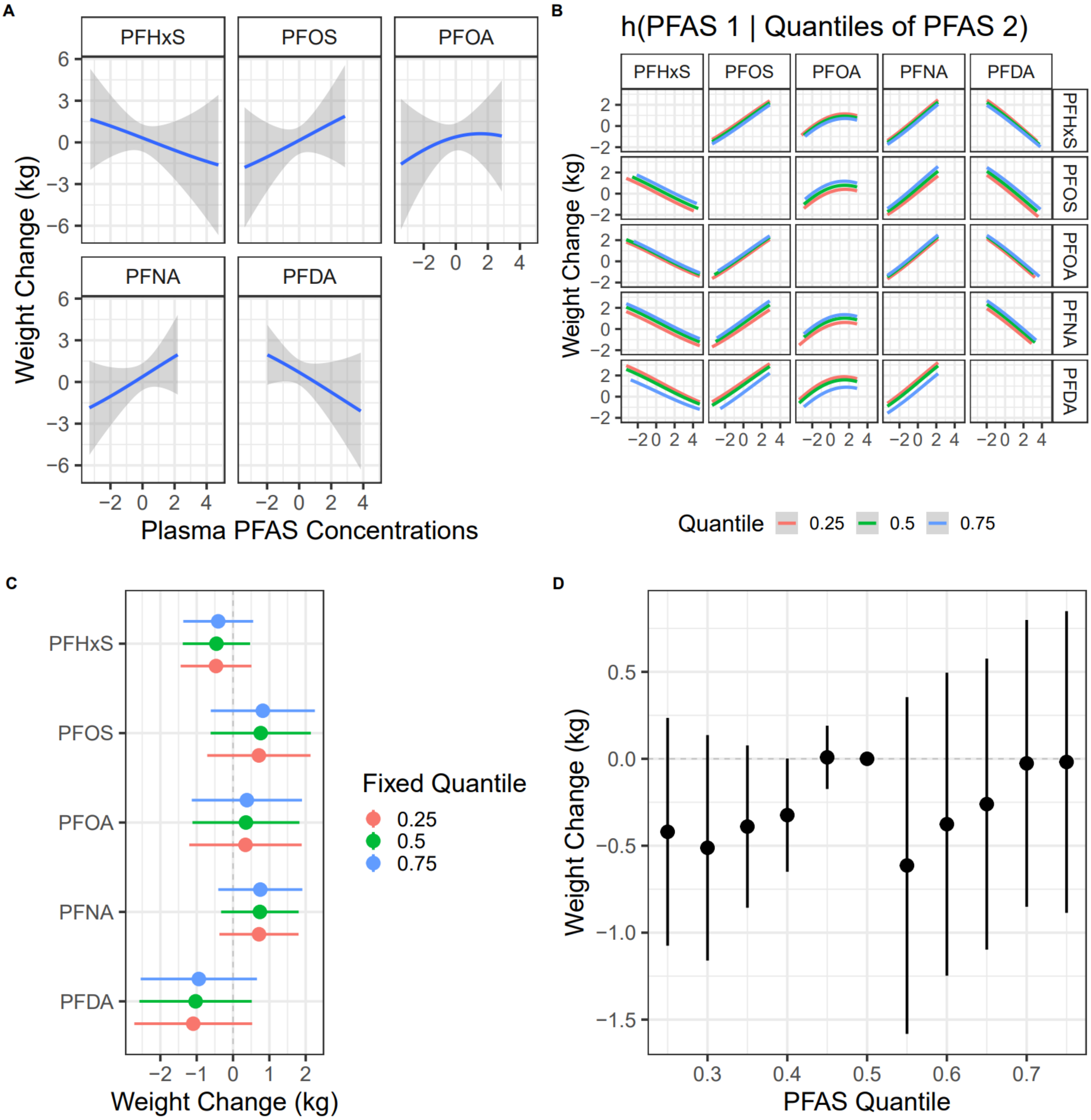

Figure S5. Associations between the PFAS mixture plasma concentrations during pregnancy and postpartum weight change (kg) in one randomly selected dataset from multiple imputation in the New Hampshire Birth Cohort Study (NHBCS) (n = 482). S5A: Associations between individual PFAS and postpartum weight change when other PFAS are fixed at their median values. S5B: Associations between individual PFAS and postpartum weight change when a second PFAS is fixed at its 25th, 50th, and 75th percentile values, and the rest of the PFAS are fixed at their median values. S5C: Associations between individual PFAS and postpartum weight change when other PFAS are fixed at their 25th, 50th, and 75th percentile values. S5D: The difference in postpartum weight change when all PFAS are simultaneously fixed at different deciles compared to their median values. PFAS concentrations were log2-transformed and centered by their means and scaled by their standard deviations.
